# Endobronchial Lipomatous Polyp: A Rare Benign Tumor of the Lung

**DOI:** 10.1155/2014/240834

**Published:** 2014-05-25

**Authors:** Salim Surani, Karen Allen, Saherish Surani, Joseph Varon

**Affiliations:** ^1^Texas A&M University, Corpus Christi, 1177 West Wheeler Avenue, Suite 1, Aransas Pass, TX 78336, USA; ^2^Bay Area Medical Center, University of North Texas, 7101 S Padre Island Drive, Corpus Christi, TX 78402, USA; ^3^Pulmonary Associates of Corpus Christi, 1177 West Wheeler Avenue, Suite 1, Aransas Pass, TX 78336, USA; ^4^The University of Texas Health Science Center, University General Hospital, 7501 Fannin Street, Houston, TX 77054, USA

## Abstract

Endobronchial lipomatous polyp is a rare nonmalignant tumor of the lung. It comprises 5% of the benign lung tumor, with the majority of benign tumors being hamartoma. Lipomatous polyp often leads to endobronchial lesion, associated with postobstructive pneumonia, hemoptysis, and atelectasis. We hereby present a case and discussion of an elderly man with endobronchial lipomatous polyp, presenting as recurrent pneumonia.

## 1. Introduction

Less than 5% of lung tumors are benign [1, 2]. The majority of fat-containing benign lung tumors are hamartomas [3], with lipomas only making up to 0.1–0.5% of all benign lung tumors [3–5]. An endobronchial lipoma, while being itself benign, can lead to other complications associated with bronchial obstruction, including atelectasis, pleural effusion, loss of lung volume, and the symptoms as hemoptysis, cough, and dyspnea [4]. We hereby present a case report of a patient with a recurrent pneumonia as evidenced on chest radiograph, as well as symptoms thereof, including cough productive of white sputum and dyspnea.

## 2. Case Report

**Figure 1:**
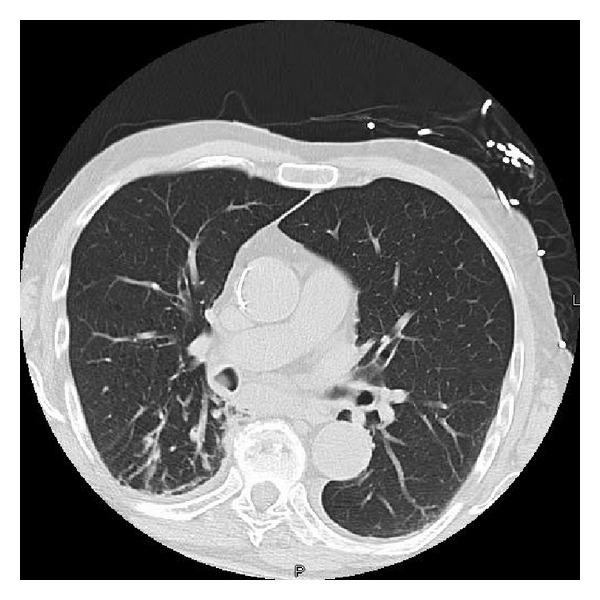
CT scan of chest showing a debris/possible endobronchial lesion in the bronchus intermedius of right lung.

**Figure 2:**
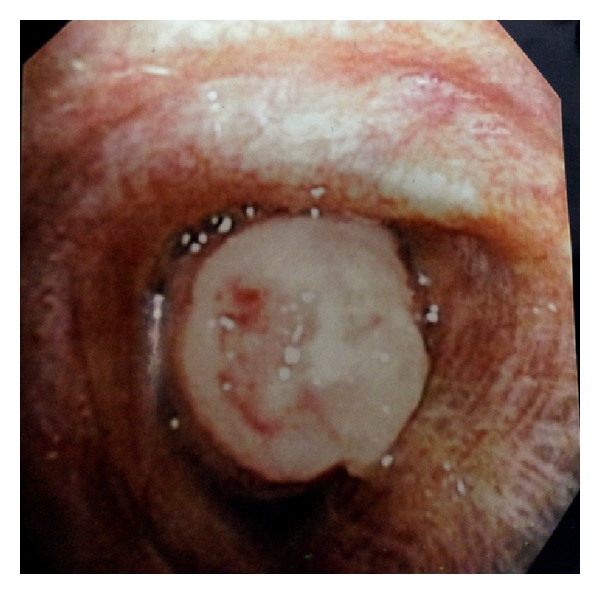
Bronchoscopic view of the lesion in the right bronchus intermedius.

**Figure 3:**
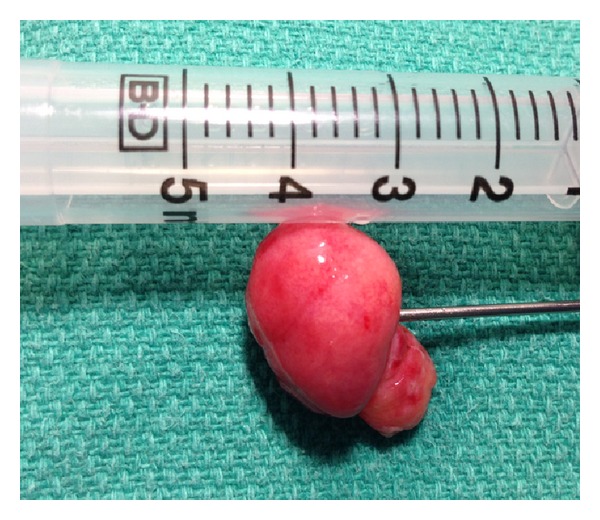
Gross appearance of the lipomatous polyp after resection by argon photocoagulation.

**Figure 4:**
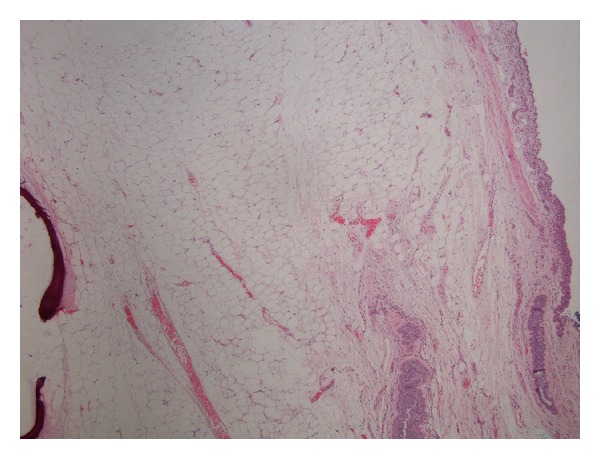
Low power H&E stain of the specimen showing respiratory epithelium, underlying fat and metaplastic bone.

**Figure 5:**
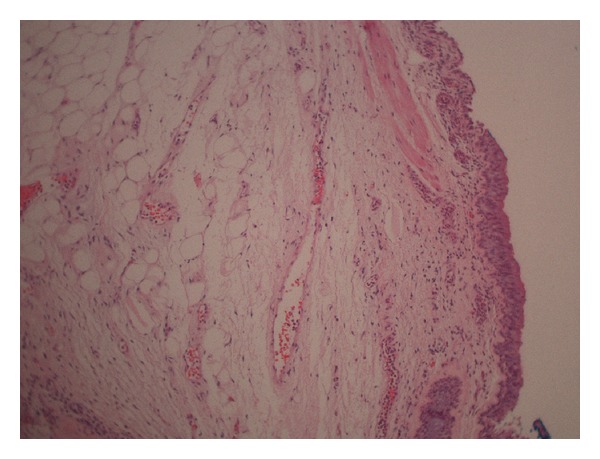
High power H&E stain showing respiratory epithelium and underlying fat.

A 79-year-old male with history of hypertension, chronic obstructive pulmonary disease (COPD), and renal cell carcinoma (treated with right nephrectomy in 2003) presented to the emergency department with the complain of low-grade temperature of 100.3° Celsius and cough for 3 days. The patient did give the history of having three bouts of pneumonia over the past year. The patient was found to have white blood cell (WBC) count of 20.5 mm^3^, hemoglobin of 13.3 grm/dL, hematocrit of 39.5, and platelet count of 572,000 *μ*L. Patient chemistry revealed mild renal insufficiency with blood urea nitrogen (BUN) of 21 mg/dL and creatinine of 1.5 md/dL. Patient chest X-ray revealed some atelectasis/consolidation of the right lower lobe. The patient underwent further evaluation with CT scan of the chest revealing chronic consolidation of the right lower lobe with some endobronchial abnormality in the right bronchus intermedius (Figure 1). The patient was admitted to hospital and was started on appropriate antibiotics. In view of some abnormality in bronchus intermedius, the patient underwent bronchoscopy, which showed the endobronchial lesion in the right bronchus intermedius, which was obstructing the entrance of right middle and lower lobes (Figure 2). Patient underwent biopsy of the lesion, which revealed benign bronchial mucosa. The patient underwent flexible fiber optic bronchoscopy and complete resection of the lesion by using argon plasma coagulation (APC), axial straight fire probe, and the snare probe. The lesion was removed in one piece (Figure 3). Pathology showed polyp covered by benign bronchial mucosa and composed primarily of adipose tissue, some area of vascularity and focal area of ossification, and chronic inflammation (Figures 4 and 5). The patient did well and was discharged home in stable condition with outpatient follow-up in two weeks. Repeated bronchoscopy in four weeks showed no residual lesion and normal mucosa using the white light flexible video bronchoscope.

## 3. Discussion

Bhatia and Ellis report that lipomatous tumors of the lung generally occur in “airways in lobar or subsegmental locations,” with peripheral pulmonary lipomas being very rare [3] and lipoma of the pulmonary parietal pleura being exceptional [6]. Muraoka et al. reviewed 64 cases of endobronchial lipomas, with a predilection for the first three tracheobronchial divisions [4], which is confirmed by Yokozaki et al. [7]. CT imaging of these tumors revealed a well-defined mass with either nodular or polypoid shape and fat attenuation, with or without calcification [3]. These descriptors on imaging are well documented in the literature, as seen in cases reported by Matsuba et al. [5], Pollefliet et al. [1], and our own case. Wood and Henderson specifically report a peripheral lipoma that was initially found on chest X-ray [8]. Because of the radiographic appearance of these nodules, they often fall under the investigative category of “solitary pulmonary nodule,” which often warrants further testing, either by serial CT imaging or biopsy [9]. Previous conventional wisdom dictated that lipomas were slow-growing and needed only distant follow-up, but some case reports have challenged this with evidence that rapid growth can occur [6]. Doulias et al. reported a peripheral pulmonary lipoma that was active on PET scan and subsequently removed by thoracotomy under suspicion of malignancy, but increased uptake on PET scan is by far the exception [10].

In most of the case reports we reviewed, the patient has undergone relatively invasive testing such as needle biopsy, bronchoscopy, or thoracotomy to rule out malignant etiology or to alleviate pulmonary complications; this tendency is also confirmed by the case review by Muraoka et al. [4] and Yokozaki et al. [7]. Of the 64 cases reviewed by Muraoka, 80% of the patients had a pathologic pulmonary process as seen on imaging, including atelectasis, volume loss, consolidation, or pleural effusion [4]. Case reports by Matsuba et al. [5] and Pollefliet et al. [1] also involved the findings of atelectasis and pleural effusion, respectively. We can attribute the predominance for radical or invasive surgery to the often destructive nature of recurrent pneumonia in patients with obstructive endobronchial lipoma [7]. Our patient received bronchoscopy because of the appearance of “debris” or questionable endobronchial lesion in his bronchus intermedius, as well as worsening pneumonia. This proved to be a fortunate course of action as we were able to remove the obstruction and relieve his symptoms before further destruction occurred. Because of the predominance for endobronchial involvement in the first few tracheobronchial divisions, bronchoscopic removal is recommended for most lipomas [4]. As with all lipomas, surgical (preferably bronchoscopic) removal is the only treatment to date to relieve the obstruction. Factors complicating the decision to proceed with bronchoscopic removal would be growth into the lung parenchyma, peripheral tumor location, or high suspicion of malignancy. Case reports mentioned in this review confirm that failure to identify the mass as a benign lesion will result in more invasive surgical removal. As with most diagnoses in medicine, complications can generally be avoided with earlier detection of the lipoma and obstruction, but, as of yet, there is no recommendation for screening as lipomatous tumors of the lung are so rare. The histology of a lipoma should, of course, include mature adipose tissue. Other histologic subtypes are sometimes present, however, including pleomorphic giant cells [5], respiratory epithelium [1], and calcification [3].

## 4. Conclusion

Lipomatous polyp is a benign lesion and bronchoscopic resection can be curative. Patient should undergo bronchoscopic evaluation either for recurrent pneumonia or if any abnormality is seen in the CT scan of the chest.
